# Resculpting the binding pocket of APC superfamily LeuT-fold amino acid transporters

**DOI:** 10.1007/s00018-017-2677-8

**Published:** 2017-10-23

**Authors:** Noel Edwards, Catriona M. H. Anderson, Nichola J. Conlon, Andrew K. Watson, Rebecca J. Hall, Timothy R. Cheek, T. Martin Embley, David T. Thwaites

**Affiliations:** Institute for Cell and Molecular Biosciences, Faculty of Medical Sciences, Newcastle University, Newcastle upon Tyne, NE2 4HH UK

**Keywords:** Amino acid, Transporter, Membrane transport, LeuT, APC superfamily, SLC

## Abstract

Amino acid transporters are essential components of prokaryote and eukaryote cells, possess distinct physiological functions, and differ markedly in substrate specificity. Amino acid transporters can be both drug targets and drug transporters (bioavailability, targeting) with many monogenic disorders resulting from dysfunctional membrane transport. The largest collection of amino acid transporters (including the mammalian SLC6, SLC7, SLC32, SLC36, and SLC38 families), across all kingdoms of life, is within the Amino acid-Polyamine-organoCation (APC) superfamily. The LeuT-fold is a paradigm structure for APC superfamily amino acid transporters and carriers of sugars, neurotransmitters, electrolytes, osmolytes, vitamins, micronutrients, signalling molecules, and organic and fatty acids. Each transporter is specific for a unique sub-set of solutes, specificity being determined by how well a substrate fits into each binding pocket. However, the molecular basis of substrate selectivity remains, by and large, elusive. Using an integrated computational and experimental approach, we demonstrate that a single position within the LeuT-fold can play a crucial role in determining substrate specificity in mammalian and arthropod amino acid transporters within the APC superfamily. Systematic mutation of the amino acid residue occupying the equivalent position to LeuT V104 titrates binding pocket space resulting in dramatic changes in substrate selectivity in exemplar APC amino acid transporters including PAT2 (SLC36A2) and SNAT5 (SLC38A5). Our work demonstrates how a single residue/site within an archetypal structural motif can alter substrate affinity and selectivity within this important superfamily of diverse membrane transporters.

**Electronic supplementary material:**

The online version of this article (doi:10.1007/s00018-017-2677-8) contains supplementary material, which is available to authorized users.

## Introduction

For any eukaryotic or prokaryotic cell to remain viable, it must express a large and diverse complement of membrane transport proteins to enable import and export, between the cell and the local environment, of all material vital for life. Carrier-mediated, transmembrane amino acid transport is essential in neurotransmission, nutrient absorption from diet, osmoregulation, and in the supply of components for protein synthesis, nitrogen metabolism, cell growth, energy production, and conversion. Thus, each cell type possesses a unique array of amino acid transporters to permit optimal physiological performance within any given milieu.

The largest collection of amino acid transporters across all forms of life is found within the Amino acid-Polyamine-organoCation (APC) superfamily [Transporter Classification DataBase (TCDB)] [1, 2]. Substrates include the 20 proteinogenic amino acids (which differ in size, shape, hydrophobicity, polarity, and charge on the α carbon side-chain), non-proteinogenic α amino acids (e.g., betaine and ornithine), and unbranched chain amino acids and analogues where the amino group is in the β or γ position (e.g., taurine, GABA). Carriers vary greatly in substrate specificity with some transporting a single type of amino acid (with extreme selectivity), others accepting almost all amino acids (with varying levels of discrimination), with most falling somewhere in between. This commonality in general function (amino acid transport), but heterogeneity in substrate selectivity, provoked this investigation into the molecular basis of carrier diversity.

The solution of the three-dimensional structure of the *Aquifex aeolicus* Na^+^/amino acid cotransporter LeuT opened a window into our understanding of APC transporter biology not only in prokaryotes but also in eukaryotes [3]. LeuT was the first APC superfamily member to be solved at atomic level [3], but others have followed including carriers of amino acids, biogenic amines, divalent metals, monosaccharides, organocations, and osmolytes [4–16]. Although these APC carriers differ in both substrate selectivity and transport mechanism (functioning as symporters or antiporters, and being driven by ionic or solute gradients), it is striking that they possess a similar structure, known commonly as the LeuT-fold [3–17]. This large APC superfamily possesses; therefore, a universal core skeleton for which the LeuT structure can be considered an archetype [18, 19].

Mammalian LeuT-fold APC transporters are also classified, based upon sequence identity, into eight solute carrier (SLC) families (SLCs 5, 6, 7, 11, 12, 32, 36, and 38), in accordance with the Human Gene Organisation (HUGO) Gene Nomenclature Committee (HGNC) [20]. Seventy-one distinct human SLCs are predicted to possess the LeuT structural-fold. They vary widely in substrate selectivity and include transporters of amino acids, sugars, neurotransmitters, vitamins, electrolytes, micronutrients, signalling molecules, and organic and fatty acids. Interpretation of the prokaryote LeuT crystal structure, relative to function, was aided extensively by the earlier structure–function studies of mammalian SLC6 transporters involved in transport of the neurotransmitters GABA, serotonin, dopamine, and noradrenaline [21–24]. In turn, near-atomic resolution structures, such as the 1.65 Å LeuT structure [3], yield great insight into the workings of distantly related mammalian solute carriers [18, 19].

Structures of LeuT-fold transporters exhibit a conserved binding environment. For example, comparison of three sequence-unrelated prokaryotic APC superfamily transporters, LeuT [from the Neurotransmitter:Sodium Symporter family (NSS, TCDB family 2.A.22)], the arginine/agmatine antiporter AdiC [from the Amino Acid-Polyamine-Organocation (APC, 2.A.3) family], and the Na^+^/benzyl-hydantoin cotransporter Mhp1 [from the Nucleobase:Cation Symporter-1 (NCS1, 2.A.39) family], demonstrate that substrates sit within the occluded structures in the same general locale [3, 9, 25]. Despite this strong structural consensus, the substrate selectivity of each carrier type remains unique.

LeuT-like structures contain a core of ten transmembrane (TM) spans, organised into a 5 + 5 inverted structural repeat, with TM1, 3, 6, and 8 forming the central binding pocket of each carrier [3–17]. In the original LeuT structure, the aliphatic side-chain of the substrate sits within a hydrophobic pocket formed from the side-chains of residues in TM3, 6, and 8 [3]. Comparative modelling of LeuT with more than 300 prokaryotic and eukaryotic NSS sequences identified that the residues that interact with the substrates’ side-chains in the deeper regions of the binding pockets are not conserved [26]. Bioinformatics analyses, using functional site prediction strategies, anticipated key functional sites within the NSS family and correctly predicted 31/34 substrate-interacting residues in the LeuT structure [27]. Residues in the three “non-predicted” positions in LeuT all form van der Waals’ contacts with the substrate side-chain [3]. LeuT was subsequently crystallised bound to a series of amino acids with increasing side-chain size [28]. When LeuT is locked in the outward-open substrate-bound conformation, by interaction with the large indole ring of the non-transported inhibitor tryptophan [28], V104 in TM3, one of the non-conserved residues identified in the bioinformatics analyses [26, 27], and the focus of the current investigation, occupies a deep position below the indole ring.

An ambition of global industry is to use in silico methodology to predict drug delivery, action of novel pharmaceuticals, and utilisation and efficiency of new agrichemicals. Ultimately, to achieve such an understanding of the roles of individual transporters in these essential functions, it is necessary to determine how the identity of amino acid residues coordinating the substrates within the binding pocket of each APC superfamily carrier defines substrate specificity. Here, we investigated the hypothesis that amino acid residues occupying the equivalent position to V104 in the LeuT-fold of APC carriers are critical in governing substrate specificity. The basis of substrate selectivity in LeuT-fold APC superfamily amino acid transporters was investigated using a series of wild-type and mutated transporters from the important Amino Acid/Auxin Permease (AAAP, 2.A.18) family [2, 29], which are expressed ubiquitously in plants, animals, yeast, and fungi. The relationship between different amino acid transporter families within the APC superfamily was investigated by computational phylogenetic methodology. Structural models were constructed based upon the outward-occluded substrate-bound conformations of the APC superfamily members LeuT, AdiC, and Mhp1 [3, 9, 25]. Site-directed mutagenesis and functional measurements of transporter activity were used to validate the structural models in multiple AAAP transporters. Excellent agreement was observed between model predictions and functional activity. In addition, re-evaluation of published data on non-amino acid transporting APC carriers suggests that the site investigated has an importance in defining substrate specificity beyond amino acid transporters. Taken together, our results demonstrate how a single residue/site within an archetypal structural motif alters substrate affinity and selectivity in an extensive, widely distributed and important superfamily of cellular transport proteins.

## Materials and methods

### Materials

Radiolabelled amino acids were from GE Healthcare Life Sciences (Little Chalfont, UK), PerkinElmer (Beaconsfield, UK), Hartmann Analytic (Braunschweig, Germany), and American Radiolabeled Chemicals (St. Louis, USA). In vitro transcription reagents were from Promega (Southampton, UK) or Ambion (Warrington, UK). Site-directed mutagenesis QuikChange Lightning kit, reagents, and primer design were from Agilent Technologies (Stockport, UK).

### Phylogeny

Sequences for the APC superfamily tree were retrieved from the TCDB [1]. An initial selection of sequences included TCDB entries (denoted TC#) with structural data plus TCDB entries with strong functional characterisation. Additional sequences were added to improve both the taxonomic diversity of the sampling and to cover additional APC families. Human sequences for SLCs 5, 6, 7, 11, 12, 32, 36, and 38 were retrieved via the Bioparadigms database [20]. Additional sequences (rat, mouse, rabbit, *Drosophila melanogaster*, *Aedes aegypti,* and *Acyrthosiphon pisum*) were from NCBI and were included for reference. All sequences were aligned using MUSCLE [30]. The alignment was trimmed using TrimAl v1.4 [31], with trimming parameters defined by the automated1 option. Phylogenies were generated in PhyloBayes [32] using the CAT20 model [33]. Trimmed (and untrimmed) sequence alignments associated with the phylogenies are available at figshare using the link: https://figshare.com/s/378479b6958df7816b1b.

### Threading, alignment, and homology modelling

HHPred [34] and Modeller [35] were used (using default settings) (http://www.toolkit/tueingen/mpg.de) to derive alignments and model eukaryotic and prokaryotic transporters based on the substrate-bound, outward-occluded, crystal structures of the following prokaryotic transport proteins: the Na^+^/amino acid cotransporter LeuT from *A. aeolicus* (bound to leucine, 1.65 Å resolution, protein data bank (PDB) ID 2A65) [3], the arginine/agmatine antiporter AdiC from *E. coli* (bound to arginine, 3.0 Å resolution, 3L1L) [9], and the Na^+^-coupled benzyl-hydantoin transporter Mhp1 from *M. liquefaciens* (bound to 5-indolylmethyl-l-hydantion, 3.4 Å resolution, 4D1A) [25]. In addition, other APC transporter structures were used including: LeuT (3F48), DAT (4XP4), AdiC (5J4I), ApcT (3GIA), and Mhp1 (2JLN) [5, 6, 12, 28]. For rat PAT2, HHPred probability scores were 95.7, 99.9, and 99.2% for predicted structural homology to LeuT, AdiC, and Mhp1, respectively, with most APC crystal structures scoring > 90%. PROMALS3D [36] was used (using default settings) for multi-alignments of crystal structure sequences with rat PAT2 and to confirm the results produced using other methods. The I-TASSER server (http://zhanglab.ccmb.med.umich.edu/) [37] was used (with default settings) for threading and modelling of the rat PAT2 sequence to generate a consensus model and to compare alignments with those produced by HHPred [34] and PROMALS3D [36]. Multiple structure–structure alignments of LeuT (2A65, 3F48), AdiC (3L1L, 5J4I), Mhp1 (2JLN, 4D1A), ApcT (3GIA), and the I-TASSER-derived PAT2 model were performed using Matt [38]. All approaches were also used to identify residues equivalent to LeuT V104 and rat PAT2 F159. Structures and models were visualised, and figures prepared, using PyMOL.

### Plasmid constructs and site-directed mutagenesis

The use of plasmid constructs for rat PAT2 (SLC36A2) and human SNAT5 (SLC38A5) in pSPORT1, and mouse PAT1 (SLC36A1) in pCRII-TOPO, has been described previously [39–41]. The *Drosophila* transporter CG1139 was purchased from the Drosophila Genomics Resource Centre (Indiana University, USA) and expressed in pGH19 (gift from G. Robertson, University of Wisconsin, USA). Site-directed mutagenesis was performed using the QuikChange Lightning kit, according to the manufacturer’s instructions. The PCR cycling parameters used were an initial 2 min incubation at 95 °C, followed by: 18 cycles at 95 °C denaturation (20 s); 68 °C annealing (10 s); 68 °C extension (30 s kb^−1^ plasmid); and a final extension at 68 °C for 5 min. Parental plasmid DNA was digested with *Dpn*I and the PCR reaction product used to transform XL-10 Gold cells. Oligonucleotides were designed using the QuikChange primer design tool. Mutations were verified by sequencing (GATC Biotech, London, UK) of the entire open reading frame.

### Functional expression in *Xenopus laevis* oocytes

Plasmid DNA was linearised by *Hind*III (PAT1, PAT2, and CG1139) or *Not*I (SNAT5), and used as a template for cRNA synthesis using the T7 mMessage mMachine kit (Thermo Fisher, Cramlington, UK) or as follows: linear DNA (< 3.5 μM) was incubated with T7 polymerase (50U), dNTPs, RNase inhibitor (160U), reaction buffer, dithiothreitol (0.1 M), BSA (10 μg), and m7G(5′)ppp(5′)G cap analogue (1 mM), for 2 h (37 °C). Template was degraded by addition of DNase (15 min, 37 °C). Female *Xenopus laevis* frogs were obtained from Xenopus Express (Haute-Loire, France) or Xenopus1 (Michigan, USA), killed in accordance with Home Office Schedule 1 directives, and oocytes isolated, essentially as described previously [40, 42]. Oocytes were de-aggregated using collagenase A (2.5 mg ml^−1^, Roche, Burgess Hill, UK) in ORII solution and washed in modified Barth’s solution. Healthy-looking stage V/VI oocytes were manually defolliculated as required and stored in modified Barth’s solution (18 °C) for approximately 24 h before being injected. Oocytes were injected with 50 nl water (control) or cRNA (1 μg μl^−1^) using a Nanoject II automated injector (Drummond Scientific Company, Broomall, USA) and maintained for 2–3 days at 18 °C until use in radiotracer transport or two-electrode voltage-clamp (TEVC) assays.

### Radiolabelled amino acid transport assays

Radiolabelled amino acid transport (uptake) assays were performed, as described previously [42]. In brief, oocytes were incubated at room temperature (approximately 22 °C) in transport solution [100 mM choline chloride (or 100 mM NaCl for solutions requiring Na^+^), 2 mM KCl, 1 mM CaCl_2_, 1 mM MgCl_2_, 10 mM MES, or HEPES adjusted to the required pH with Tris base] containing [^3^H]- or [^14^C]-labelled compound (1–5 μCi ml^−1^). Assays were performed at pH 5.5, in the absence of extracellular Na^+^, unless stated otherwise. Oocytes were then washed three times in ice-cold transport solution and lysed in 10% SDS, and the associated radioactivity determined by liquid scintillation counting.

### Two-electrode voltage clamp (TEVC)

Individual oocytes, which had been injected with either CG1139 cRNA or water (control), were clamped at − 60 mV and superfused with Na^+^-free, pH 5.5 transport solution (see above), as described previously [42]. Various amino acids were added for 1 min and the associated inward positive current measured using a Geneclamp 500 amplifier, Digidata 1200, and pClamp software (Molecular Devices, Sunnyvale, CA). Compound-associated currents were calculated by averaging the current during the last 15 s of exposure and subtracting the average current recorded in the 15 s preceding exposure (baseline). Data were analysed using Clampfit 8.2.

### Data and statistical analysis

Data are mean ± SEM. and are typically expressed as pmol.oocyte^−1^ (uptake duration)^−1^. Transporter-specific uptake was calculated as uptake into transporter-expressing oocytes after subtraction of uptake into water-injected oocytes (measured under identical conditions). Michaelis–Menten kinetics were fitted using GraphPad Prism 6. Comparisons of mean values were made by one-way or two-way analysis of variance (ANOVA), as appropriate, with Tukey’s or Sidak’s multiple comparisons post-tests (GraphPad Prism 6). ANOVAs are two-way unless stated otherwise.

## Results

### LeuT-fold transporters are highly divergent in their overall amino acid sequences

**Fig. 1:**
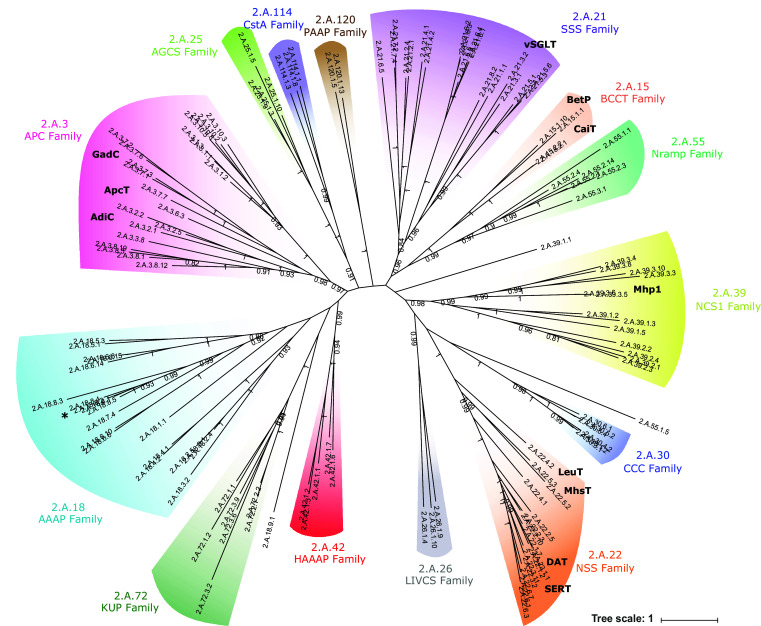
Phylogenic analysis of the APC superfamily including representative sequences from all major APC families whose transport proteins are predicted to possess the LeuT-fold 5 + 5 inverted structural repeat. The 14 families are the: Amino Acid-Polyamine-Organocation (APC) family (TC# 2.A.3), which includes the mammalian SLC7 family; Betaine/Carnitine/Choline Transporter (BCCT) family (2.A.15); Amino Acid/Auxin Permease (AAAP) family (2.A.18), including mammalian SLC32, SLC36 and SLC38 families; Solute:Sodium Symporter (SSS) family (2.A.21), including the mammalian SLC5 family; Neurotransmitter:Sodium Symporter (NSS) family (2.A.22), including the mammalian SLC6 family; Alanine or Glycine:Cation Symporter (AGCS) family (2.A.25); Branched Chain Amino Acid:Cation Symporter (LIVCS) family (2.A.26); Cation-Chloride Cotransporter (CCC) family (2.A.30), including the mammalian SLC12 family; Nucleobase:Cation Symporter-1 (NCS1) family (2.A.39); Hydroxy/Aromatic Amino Acid Permease (HAAAP) family (2.A.42); Metal Ion (Mn^2+^-iron) Transporter (Nramp) family (2.A.55), including the mammalian SLC11 family; K^+^ Uptake Permease (KUP) family (2.A.72); Putative Peptide Transporter Carbon Starvation CstA (CstA) family (2.A.114); Putative Amino Acid Permease (PAAP) family (2.A.120). The position of PAT2 (TC# 2.A.18.8.2) is indicated by asterisk. Example transporters with known atomic structures (included in the TCDB) are denoted: AdiC of *E. coli* (2.A.3.2.5); ApcT of *M. jannaschii* (2.A.3.6.3); GadC of *E. coli* (2.A.3.7.3); BetP of *C. glutamicum* (2.A.15.1.10); CaiT of *E. coli* (2.A.15.2.1) and *P. mirabilis* (2.A.15.2.2); vSGLT of *V. parahaemolyticus* (2.A.21.3.2); SERT of *H. sapiens* (2.A.22.1.1); DAT of *D. melanogaster* (2.A.22.1.7); LeuT of *A. aeolicus* (2.A.22.4.2); MhsT of *B. halodurans* (2.A.22.5.3); and Mhp1 of *M. liquefaciens* (2.A.39.3.6). In addition, there are several crystals from the Nramp (2.A.55) family (not shown). DraNramp (MntH) of *D. radiodurans* (2.A.55.3.7) has recently been added to the TCDB. The phylogeny was generated using the CAT20 model in PhyloBayes. Each branch, representing an individual transporter sequence, is identified by the appropriate TCDB number (see list of sequences in supplementary Table S1). Values at branches represent posterior probabilities (scale bar: average number of substitutions per site). The majority of APC families are well supported as clans. Three sequences branch separately from their annotated family but are located close to the base of the tree with very weak support, suggesting that their position is an artefact due to long-branch attraction

The APC superfamily consists of 18 transporter families [2], 14 of which are predicted to possess the LeuT-fold 5 + 5 inverted structural repeat. Phylogenetic sequence analysis of prokaryotic and eukaryotic representative members of the fourteen families indicates that most are well supported as putative monophyletic groups or clans [43] (Fig. 1, supplementary Table S1). The relationships between individual families, however, are generally poorly supported highlighting the divergent nature of the superfamily as a whole (which is also reflected in their differing functions). Six of the fourteen families (APC, 2.A.3; AAAP, 2.A.18; AGCS, 2.A.25; LIVCS, 2.A.26; HAAAP, 2.A.42; PAAP, 2.A.120) exclusively contain amino acid transporters. Three families (BCCT, 2.A.15; SSS, 2.A.21; NSS, 2.A.22) contain amino acid transporters as well as carriers of other substrates. The remaining five families contain only other (non-amino acid transporting) carriers. Examples of transporters of known structure in the APC, SSS, BCCT, NCS1, and NSS families [3–12, 14, 15] are denoted in bold in Fig. 1 (see also supplementary Table S1). There appears to be no strong relationship between function and phylogeny underlining the importance of investigating sequence, structure and function, in an integrated manner to understand transport specificity. To investigate the importance of the LeuT V104-equivalent residue as a molecular determinant of substrate specificity in the APC superfamily we chose, therefore, an exemplar amino acid carrier with which to begin our analyses. The mammalian proton/amino acid cotransporter PAT2 (SLC36A2) [39, 40] (asterisk in Fig. 1, see also Table 1) is a member of the Amino Acid/Auxin Permease (AAAP, 2.A.18) family [2, 29] of transporters which are found in plants, animals, yeast, and fungi. PAT2 was chosen to sample an area of the phylogenic tree which has, to date, been underexplored and to investigate the generality of the observations in relation to the APC superfamily as a whole. PAT2 is a tractable transport protein, amenable to mutagenesis and functional measurements. It has a narrow and well-defined substrate selectivity that appears to be restricted severely by side-chain size [39, 40], identifying it as a suitable candidate for functional and mutational analyses. In humans, PAT2 contributes to amino acid transport in diverse cell types such as renal proximal tubule cells, neurones, and adipocytes. Mutations in PAT2 leading to defective function contribute to the human disorders of iminoglycinuria (Online Mendelian Inheritance in Man (OMIM) 242600) and hyperglycinuria (OMIM 138500) [44].

**Table 1:** APC superfamily transporters (as discussed in this paper)

Transporter (common name)	Species or class	SLC gene name	TCDB number(s)*	Example PDB number(s) (if crystallised)	Predicted LeuT V104-equivalent residue	Substrate class
2.A.18 Amino acid/auxin permease (AAAP) family						
PAT2	Mammalia	SLC36A2	2.A.18.8.2 (mouse) 2.A.18.8.6 (human)		F159 (rat) F161 (human)	Amino acid
CG1139	*D. melanogaster*				I149	Amino acid
AaePAT1	*A. aegypti*				T167	Amino acid
ApGLNT1	*A. pisum*				C198	Amino acid
SNAT5	Mammalia	SLC38A5	2.A.18.6.8 (rat) 2.A.18.6.15 (human)		A138 (human)	Amino acid
2.A.22 Neurotransmitter:sodium symporter (NSS) family						
LeuT	*A. aeolicus*		2.A.22.4.2	2A65, 3F48	V104	Amino acid
DAT	*D. melanogaster*		2.A.22.1.7	4XP4	V120	Monoamine
DAT	Mammalia	SLC6A3	2.A.22.1.3 (human)		V152 (human)	Monoamine
SERT	Mammalia	SLC6A4	2.A.22.1.1 (human)	5I6X	I172 (human)	Monoamine
NET	Mammalia	SLC6A2	2.A.22.1.2 (human)		V148 (human)	Monoamine
CT1	Mammalia	SLC6A8	2.A.22.3.11 (human) 2.A.22.3.5 (rat) 2.A.22.3.4 (rabbit)		C144 (human)	Creatine
2.A.3 Amino acid-polyamine-organocation (APC) family						
AdiC	*E. coli*		2.A.3.2.5	3L1L, 5J4I	G100	Amino acid
ApcT	*M. jannaschii*		2.A.3.6.3	3GIA	S100	Amino acid
LAT2	Mammalia	SLC7A8	2.A.3.8.6 (rat) 2.A.3.8.20 (human)		N133 (mouse) N134 (human)	Amino acid
LAT1	Mammalia	SLC7A5	2.A.3.8.1 (rat) 2.A.3.8.25 (human)		S144 (human)	Amino acid
2.A.39 Nucleobase:cation symporter-1 (NCS1) family						
Mhp1	*M. liquefaciens*		2.A.39.3.6	4D1A, 2JLN	W117	Hydantoin
2.A.30 Cation-chloride cotransporter (CCC) family						
NKCC1	Mammalia	SLC12A2	2.A.30.3.1 (human)		A379 (human)	Electrolyte

* The TCDB database does not currently list all characterized transporters but includes many examples from different families

### Identification of the LeuT V104-equivalent in the AAAP and SLC36 transporter PAT2

**Fig. 2:**
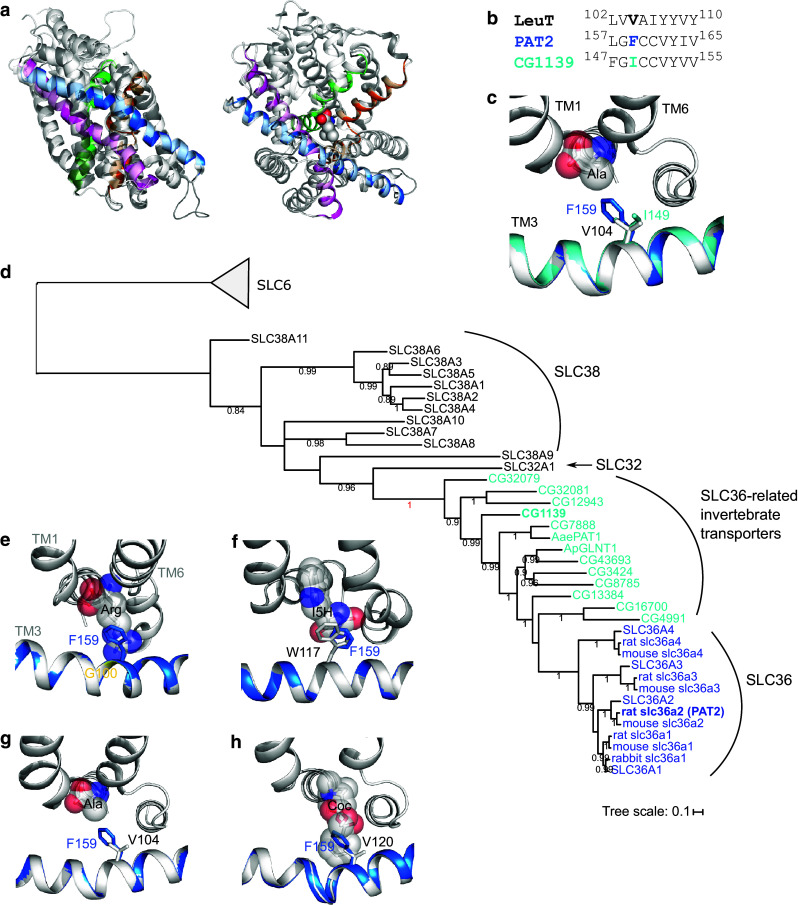
Homology modelling predicts that the SLC36 transporter PAT2 is a LeuT-fold protein and F159 is equivalent to LeuT V104. **a** PAT2 modelled against the substrate-bound, outward-occluded crystal structure (light grey ribbons) of LeuT bound to leucine (PDB ID 2A65). Structures are shown from both side-on, within the plane of the membrane (left-hand column), and top-down, above the plane of the membrane (right-hand column), orientations. The predicted PAT2 structures are represented as ribbons in dark grey and in colour. The substrate-binding pocket of PAT2 is predicted to be formed by TM1 (green), TM3 (blue), TM6 (orange), and TM8 (magenta). The comparative models include only TM1-10 (the core 5 + 5) with transmembrane domains outside the core LeuT-fold omitted. The substrate, leucine, bound in the crystal structures, is shown as spheres. **b** HHPred alignment of part of TM3 in LeuT, rat PAT2, and *D. melanogaster* protein CG1139. **c** Crystal structure of LeuT (light grey) as ribbons with the predicted structures of TM3 from rat PAT2 (blue) and CG1139 (cyan) superimposed. The side-chains of V104 (LeuT), F159 (PAT2) and I149 (CG1139) are shown as sticks. Alanine is shown as a substrate (spheres) and was superposed upon the leucine backbone bound to the crystal (2A65) using PyMOL. The PAT2 F159 (blue) and CG1139 I149 (cyan) residues are predicted to restrict the volume of the substrate-binding pocket. **d** Phylogenetic tree, generated using the CAT20 model in PhyloBayes, showing a cluster of SLC36-related invertebrate transporters including CG1139 from *D. melanogaster.* The tree includes all human, and some rodent, AAAP (2.A.18) family transporters: SLC36 (blue, rat PAT2 shown in bold); SLC32 (which has only one human member); SLC38 (all human members included). Invertebrate transporters (cyan) shown are the 11 *D. melanogaster* sequences (starting CG, CG1139 shown in bold), and the functionally characterized amino acid transporters: AaePAT1 from *A. aegypti* and ApGLNT1 from *A. pisum* (Table 1). The tree is outgroup-rooted to the human SLC6 family (part of the NSS (2.A.22) family within the APC superfamily). Values at branches represent posterior probabilities (scale bar: average number of substitutions per site). **e**–**h** Homology modelling predicts that PAT2 F159 occupies a structurally homologous position in the binding pocket when modelled upon different LeuT-fold transporters. Homology models of PAT2 TM3 (blue ribbons, with F159 shown as blue sticks) superimposed upon the substrate-bound crystal structures (all light grey ribbons) of **e** arginine (Arg)-bound AdiC (3.0Å resolution, 3L1L), **f** 5-indolylmethyl-l-hydantion (I5H)-bound Mhp1 (3.4 Å resolution, 4D1A), **g** alanine (Ala)-bound LeuT (1.90 Å resolution, 3F48), and **h** cocaine (Coc)-bound DAT (2.80 Å resolution, 4XP4), demonstrate that PAT2-F159 occupies a similar position in transmembrane domain 3 (TM3) to **e** AdiC G100 (yellow ribbon), **f** Mhp1 W117 (grey sticks), **g** LeuT V104 (grey sticks), and **h** DAT V120 (grey sticks)

The LeuT V104-equivalent residue in PAT2 was identified by homology modelling and multi-alignment. Consistent results were obtained using a number of programmes and APC transporter structures, from different families (Fig. 1), as templates (see below and Methods for details). Since a substrate’s “best-fit” will likely be represented by the outward-occluded, substrate-bound, conformation of a carrier, PAT2 was initially superimposed on the outward-occluded, substrate-bound crystal structures of LeuT [protein data bank (PDB) ID 2A65] [3], AdiC (3L1L) [9], and Mhp1 (4D1A) [25] using HHPred and Modeller [34, 35] (e.g., Fig. 2a). LeuT V104 (TM3) aligns with the larger, aromatic amino acid F159 in rat PAT2 (TM3) (Fig. 2b). F159 (rat PAT2) is equivalent to F161 in human PAT2 (Table 1). LeuT V104 and PAT2 F159 overlap within the crystal and predicted structure (Fig. 2c). PAT2 F159 also aligns with, and occupies the same locality, as AdiC G100, Mhp1 W117, and V104 in the alanine-bound occluded LeuT structure (3F48) [28], V120 in the outward-open, cocaine-bound, dopamine transporter (DAT) (4XP4) [45] (Fig. 2e–h), and S100 in the amino acid transporter ApcT (3GIA, not shown) [6] (Table 1). Eight of nine threading programmes used by I-TASSER [37] align PAT2 F159 with the same residues in the APC crystal structures as HHPred. Multi-alignment using PROMALS3D [36] identified that V104 (LeuT, 2A65), G100 (AdiC, 3L1L), W117 (Mhp1, 4D1A), V120 (DAT, 4XP4), and S100 (ApcT, 3GIA) align with each other and also with F159 in PAT2. The top PAT2 model (by cluster size) in I-TASSER had a C score of -1.87 and a TM score of 0.49 (scores improved to − 0.83 and 0.61 if only the core 5 + 5, TM1-10 inclusive, sequence was submitted) [37]. Multiple pairwise structure–structure alignments in Matt [38] identified that F159 (in the I-TASSER top PAT2 model), V104 (LeuT), G100 (AdiC), and W117 (Mhp1), were in equivalent positions, consistent with the predictions from I-TASSER, HHPred/Modeller, and PROMALS3D.

To investigate if, and how, the identity of the amino acid at the equivalent position to V104 in the LeuT-fold affects substrate selectivity in APC superfamily transporters, species-scanning mutagenesis [23] was used. In this approach, a residue is switched to the equivalent residue of a homologous transporter from a second species and the effect on transport function determined. The *Drosophila melanogaster* transporter CG1139 is found in a cluster of SLC36-related transporters (Fig. 2d). CG1139 has 37% sequence identity to rat PAT2 (using MUSCLE [30]) making it an appropriate comparator for this species-scanning approach as it is likely to retain overall mode of function (H^+^/amino acid cotransport) [46]. The other AAAP mammalian families (SLC32 and SLC38) are more distantly related (24% and 20–24% identity to PAT2, respectively) (Fig. 2d) and differ in function to the SLC36 carriers. Homology modelling identifies that CG1139 has an isoleucine at position 149 which occupies the equivalent position to PAT2 F159 (and LeuT V104) (Fig. 2b, c, Table 1).

### The *Drosophila melanogaster* transporter CG1139 has broader substrate specificity than mammalian SLC36 carriers

The transport of proteinogenic α amino acids via PAT2 is limited to proline, glycine, and alanine [39, 40, 42, 47, 48]. CG1139 was previously shown to transport alanine (inhibited by glycine and proline) [49]. When modelled upon the LeuT amino acid-bound structure, CG1139 I149 occupies the same position as PAT2 F159 and LeuT V104 (Fig. 2b, c). The reduced volume [50] of the I149 side-chain (relative to F159) identifies CG1139 as a suitable model transporter for comparison with PAT2 for the investigation of substrate selectivity. The difference in volumes occupied by the two residues led us to predict that CG1139 would transport α amino acids with larger side-chains than those able to access PAT2.

**Fig. 3:**
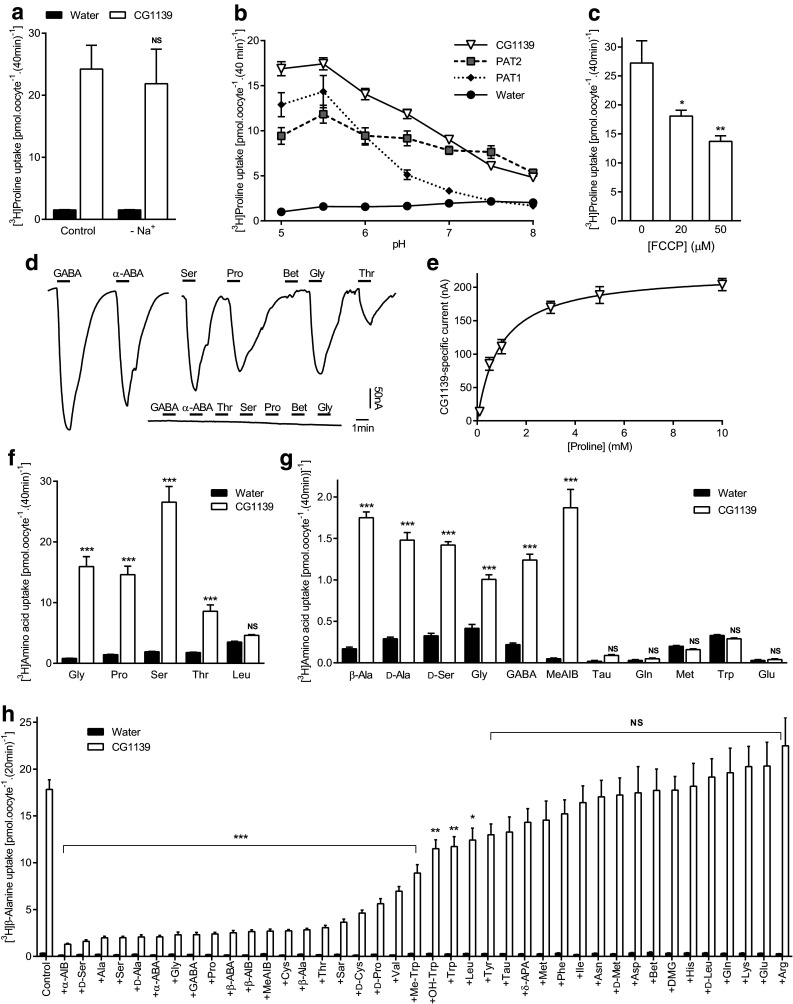
Invertebrate APC transporter CG1139 is a pH-dependent, Na^+^-independent, amino acid transporter with differing substrate specificity to the mammalian SLC36 transporters. **a** Proline (10 μM) uptake (extracellular pH 5.5) in the presence (control) and absence (-Na^+^) of extracellular Na^+^ in oocytes injected with CG1139 cRNA. Uptake in water-injected oocytes is shown as a control. *n* = 10; NS (not significant), *P* > 0.05 vs. control (ANOVA, Tukey’s multiple comparisons test). **b** Proline (10 μM) uptake via CG1139, PAT1, and PAT2 at varying extracellular pH in the absence of extracellular Na^+^. *n* = 9–10. **c** CG1139-mediated proline (10 μM) uptake (pH 5.5) in the absence and presence of the protonophore FCCP (20–50 µM). *n* = 10; *, *P* < 0.05; **, *P* < 0.01 vs. zero FCCP (one-way ANOVA, Tukey’s multiple comparisons test). **d** Amino acid-induced inward currents during superfusion with extracellular amino acids (all 10 mM, pH 5.5, Na^+^-free conditions) in two-electrode voltage-clamped oocytes (clamped at − 60 mV) injected with either CG1139 cRNA (main trace) or water as a control (inset) (representative of 5 and 4 experiments, respectively). ABA, aminobutyric acid; Bet, betaine. **e** Concentration-dependent, proline-induced, CG1139-mediated inward current. Data represent current elicited by proline (0.1–10 mM, 1 min) in CG1139-expressing oocytes after subtraction of that in water-injected oocytes under identical conditions. *n* = 4. **f**, **g** Radiotracer amino acid uptake [10 μM (**f**) and 400 nM (**g**)] via CG1139 compared to control (water-injected oocytes). All amino acids are l-isomers unless stated otherwise. MeAIB, α(methylamino)isobutyric acid; Tau, taurine. *n* = 9–10; NS, *P* > 0.05; ***, *P* < 0.001 vs. water (ANOVA, Sidak’s multiple comparisons test). **h** β-Alanine (10 μM) uptake measured in the absence (control) and presence of amino acids or analogues (all 10 mM except Tyr which is 2.5 mM). All amino acids are l-isomers unless stated otherwise. AIB, aminoisobutyric acid; Sar, sarcosine; Me-Trp, α-methyl-d,l-tryptophan; OH-Trp, 5-hydroxy-l-tryptophan; APA, aminopentanoic acid; DMG, dimethylglycine. *n* = 18–20; NS, *P* > 0.05; *, *P* < 0.05; **, *P* < 0.01; ***, *P* < 0.001 vs. control (one-way ANOVA, Sidak’s multiple comparisons test whereby all bars were compared to control for CG1139 only)

CG1139-mediated amino acid transport was first characterized by several complementary measurements (Fig. 3) with the essential characteristics being similar to mammalian PAT1 and PAT2 transporters [39, 40, 42, 47, 51]. CG1139 H^+^/amino acid transport was Na^+^-independent (Fig. 3a), pH-dependent (Fig. 3b), H^+^ gradient-dependent [reduced by FCCP (Fig. 3c)], rheogenic (Fig. 3d, e), and saturable (two-electrode voltage clamp (TEVC) Km = 0.97 ± 0.15 mM for proline (Fig. 3e), and radiotracer proline uptake Km = 1.03 ± 0.18 mM).

CG1139 transports the prototypical SLC36 proteinogenic substrates (alanine, proline, and glycine) and shares other SLC36-like characteristics (Fig. 3). The striking difference shown here is that CG1139 also accepts l-amino acids with longer side-chains including serine, cysteine, and α-aminobutyric acid (α-ABA) (Fig. 3d, f–h). To test whether F159 in PAT2 limits access of extended substrate side-chains compared to the less bulky CG1139 I149 (Fig. 2c), we replaced F159 in PAT2 with isoleucine.

### The residue occupying position 159 in PAT2 determines accessibility of the substrate side-chain within the binding pocket

**Fig. 4:**
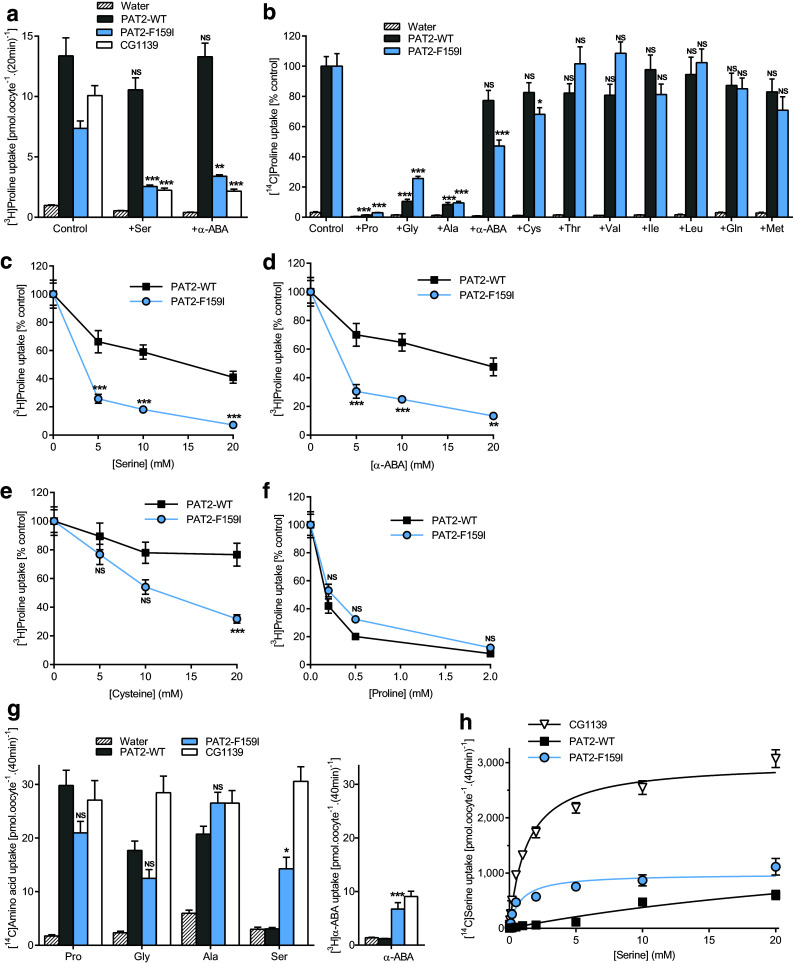
Substitution of F159 in PAT2 with isoleucine, the equivalent residue in CG1139, enables PAT2-F159I to transport amino acids with longer side-chains. Amino acid uptake was measured in oocytes expressing wild-type PAT2 (PAT2-WT), PAT2 with the F159I mutation (PAT2-F159I), or CG1139. Uptake was also measured in oocytes injected with water as a control. **a** Proline (10 μM) uptake in the absence (control) or presence of serine or α-ABA (both 5 mM). *n* = 19–20; NS, *P* > 0.05; **, *P* < 0.01; ***, *P* < 0.001 vs. control (ANOVA, Tukey’s multiple comparisons test). **b** Proline (10 μM) uptake in the absence (control) or presence of various unlabelled amino acids (all 10 mM). Data are expressed as % control (that in the absence of unlabelled amino acid). Uptake into water-injected oocytes is expressed as % PAT2-F159I control. *n* = 17–20; NS, *P* > 0.05; *, *P* < 0.05; ***, *P* < 0.001 vs. control (ANOVA, Tukey’s multiple comparisons test). **c–f** Proline uptake in the presence of **c** serine, **d** α-ABA or **e** cysteine (all 0–20 mM), or **f** unlabelled proline (0–2 mM). Data are expressed as % control (absence of competitor) after the subtraction of uptake into water-injected oocytes. *n* = 18–20; NS, *P* > 0.05; **, *P* < 0.01; ***, *P* < 0.001 vs. PAT2-WT (ANOVA, Sidak’s multiple comparisons test). **g** Uptake of various [^3^H/^14^C] amino acids (all 10 μM). *n* = 27–30 except α-ABA (*n* = 20); NS, *P* > 0.05; *, *P* < 0.05; ***, *P* < 0.001 vs. PAT2-WT (ANOVA, Tukey’s multiple comparisons test). **h** Concentration-dependent serine uptake (0.01–20 mM) by CG1139, PAT-WT, and PAT2-F159I, after subtraction of uptake into water-injected oocytes measured under identical conditions. *n* = 18–20

The large aromatic phenylalanine in PAT2 was replaced with the equivalent but smaller isoleucine from CG1139 (Figs. 2, 4). Like CG1139, but not wild-type PAT2, competition experiments demonstrate that serine, α-ABA and cysteine can now access the binding pocket and inhibit PAT2-F159I transport (Fig. 4a–e). Larger side-chains are excluded from both PAT2 and PAT2-F159I (Fig. 4b). The selectivity change in PAT2-F159I is due to improved affinity for serine, α-ABA, and cysteine (all *P* < 0.001), whereas there is a consistent but insignificant decrease (*P* = 0.054) in affinity for proline (Fig. 4c–f). PAT2-F159I not only binds Ser and α-ABA but efficiently translocates these amino acids (Fig. 4g). In PAT2, proline, glycine, and alanine have Km values in the range 120–700 μM [39, 40, 42], whereas serine is a very weak substrate. In contrast, the PAT2-F159I Km for serine (823 ± 202 μM) is close to that of CG1139 (Km 1.31 ± 0.12 mM) (Fig. 4h). This change in PAT2 selectivity, following F159I mutation to become CG1139-like (Figs. 3, 4), demonstrates clearly that residue size at position 159 is a key determinant of the substrate side-chain that can fit within the SLC36 family-binding pocket.

**Fig. 5:**
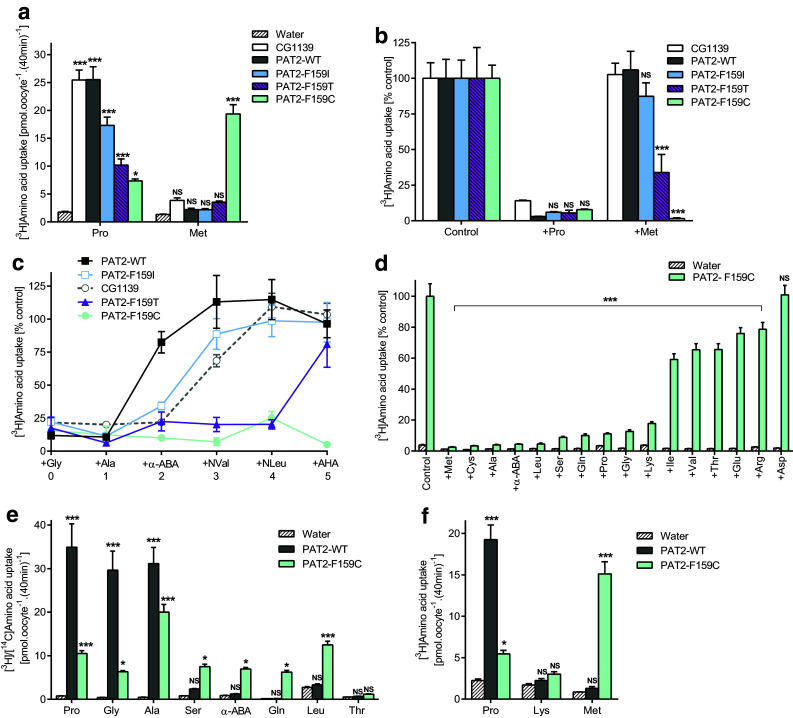
Reducing the side-chain volume of the binding pocket residue (F159) in PAT2 by substitution to cysteine (F159C) produces a gain-of-function methionine transporter. Amino acid uptake was measured in oocytes expressing wild-type PAT2 (PAT2-WT), PAT2 with either the F159I, F159T, or F159C mutations, or CG1139. Uptake was also measured in oocytes injected with water as a control. **a** Proline and methionine (both 10 μM) uptake. *n* = 18–20; NS, *P* > 0.05; *, *P* < 0.05; ***, *P* < 0.001 vs. water (ANOVA, Tukey’s multiple comparisons test). **b** Amino acid uptake (proline (10 μM) for PAT2-WT, PAT2-F159I, PAT2-F159T, and CG1139; methionine (10 μM) for PAT2-F159C only) measured in the absence (control) or presence of excess proline or methionine (10 mM). Uptake in water-injected oocytes has been subtracted. *n* = 10; NS, *P* > 0.05; ***, *P* < 0.001 vs. PAT2-WT (ANOVA, Tukey’s multiple comparisons test). **c** Uptake of proline (10 μM) or methionine (10 μM, PAT2-F159C only) measured in the absence (control) or presence of amino acids or analogues of increasing side-chain length (all 10 mM). Uptake in water-injected oocytes has been subtracted. Numbering on the *x*-axis indicates the number of side-chain carbons within the competitor compound. NVal, norvaline (2-aminopentanoic acid); NLeu, norleucine (2-aminohexanoic acid); AHA, 2-aminoheptanoic acid. *n* = 9–10. **d** [^3^H]Amino acid (10 μM) uptake by PAT2-F159C measured in the absence (control) or presence of excess, unlabelled amino acids (all 10 mM). *n* = 17–20; NS, *P* > 0.05; ***, *P* < 0.001 vs. control (one-way ANOVA, Tukey’s multiple comparisons test whereby all bars were compared to control for PAT2-F159C only). **e** Uptake of various amino acids (all 10 µM, extracellular pH 5.5, Na^+^-free conditions) into oocytes injected with wild-type PAT2 (PAT2-WT), PAT2-F159C, or water (as a control). *n* = 10. NS, *P* > 0.05; *, *P* < 0.05; ***, *P* < 0.001 vs. water (ANOVA, Tukey’s multiple comparisons test). **f** Proline, lysine, and methionine uptake (all 10 μM) measured into wild-type PAT2, PAT2-F159C or water-injected oocytes. *n* = 19–20. NS, *P* > 0.05; *, *P* < 0.05; ***, *P* < 0.001 vs. water (ANOVA, Tukey’s multiple comparisons test)

**Fig. 6:**
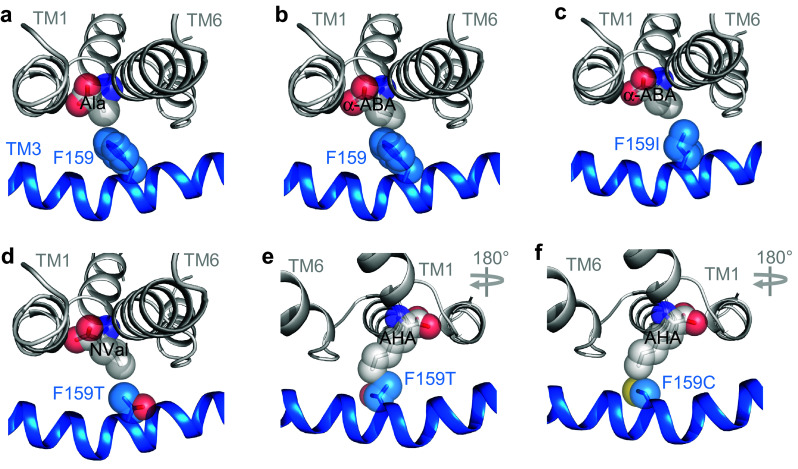
Substitution of PAT2 F159 with smaller side-chain residues is predicted to enlarge the substrate-binding pocket and concomitantly increase access of substrates with elongated side-chains. Predicted substrate-binding pocket of PAT2 modelled against the outward-open substrate-bound LeuT structure (2A65). Parts of TM1 (grey), TM6 (grey), and TM3 (blue) are shown as ribbons with potential substrates presented as spheres. The side-chain of F159 and related mutants are shown as blue spheres (with the oxygen atom of threonine in red and the sulphur atom of cysteine in yellow). **a**, **b** Prototypical PAT2 substrate alanine (Ala) and the amino acid analogue α-ABA, which is excluded from PAT2, are shown in the binding pocket of wild-type PAT2. Alanine fits within the pocket, but extension of the side-chain by the single methylene group in α-ABA produces a clash with the large volume aromatic ring of F159 thus limiting access. **c** Substitution of F159 with the smaller side-chain of isoleucine (F159I) permits α-ABA access to the PAT2 substrate-binding pocket. **d**, **e** Substitution of F159 with threonine (F159T) is predicted to further increase the PAT2 substrate-binding pocket to accommodate NVal but not the side-chain extended AHA which clashes with the threonine side-chain. **f** Access of AHA to the PAT2 substrate-binding pocket is permitted by a further reduction in the residue side-chain volume by substitution of F159 with cysteine (F159C), although some rearrangement of the flexible side-chain is likely required to allow a comfortable fit. Note that the view of the PAT2 substrate-binding pocket in **a-d** has been rotated by 180º in the horizontal plane in **e** and **f** to highlight the predicted clash between the side-chains of AHA and F159T. All amino acid substrates and analogues were inserted into the binding pocket upon the leucine backbone (2A65) using PyMOL

A series of PAT2 mutants, where residue 159 was systematically reduced in size (phenylalanine 191.9 Å^3^, isoleucine 163.9 Å^3^, threonine 121.5 Å^3^, and cysteine 103.3 Å^3^) [50], retained SLC36-like characteristics, transporting proline (the gradual decrease in uptake reflecting a decrease in affinity), glycine, and alanine (Fig. 5a–e). Methionine is not transported by CG1139, PAT2, or PAT2-F159I (Fig. 5a, f). The increase in hydrophobic-binding pocket volume in PAT2-F159T allows methionine to inhibit amino acid transport (Fig. 5b) without undergoing transport (Fig. 5a), whereas the additional space in PAT2-F159C creates a gain-of-function phenotype with excellent methionine transport (Fig. 5a, f). Titration of the binding pocket volume versus substrate side-chain size was accomplished using a series of hydrocarbon side-chain extended amino acid derivatives from the simplest amino acid glycine (no side-chain) to those where the side-chain terminal carbon atom is in the beta (Ala), gamma (α-ABA), delta (norvaline, NVal), epsilon (norleucine, NLeu), and zeta (2-aminoheptanoic acid, AHA) positions (Fig. 5c). Glycine and alanine interact with all five carriers (Fig. 5c). α-ABA is excluded from PAT2 (Fig. 5c). NVal and NLeu are excluded from CG1139, PAT2, and PAT2-F159I, but they inhibit amino acid transport via PAT2-F159T and PAT2-F159C. AHA, containing the longest side-chain, can only inhibit amino acid transport by the largest binding pocket (PAT2-F159C) (Fig. 5c). For natural proteinogenic amino acids, the F159C mutation converts PAT2 from a carrier with limited space, within the binding pocket region associated with the substrate side-chain, to one that can transport longer amino acids such as methionine, glutamine and leucine (Fig. 5a, e–f). The general SLC36/PAT2 pocket mitigates against branching on the β carbon and this is retained in PAT2-F159C (relatively weak interaction with isoleucine, valine, and threonine) (Fig. 5d, e). PAT2-F159C allows access of lysine (with an epsilon-amino group and a nitrogen atom in the zeta position) into the binding pocket (Fig. 5d). However, the severely reduced rate of transport, compared to proline and methionine (Fig. 5f), suggests that the charged side-chain is incompatible with translocation. A visual summary of the comprehensive transport measurements described (Figs. 3, 4, 5) is presented in Fig. 6.

Our results suggest that the residue occupying the equivalent position to F159 (PAT2) is key to determining substrate selectivity in the SLC36 family and related invertebrate transporters (Figs. 3, 4, 5). To test whether the role of this residue is a common feature across APC superfamily amino acid transporters, the investigation was broadened. The basis of substrate selectivity was investigated in a distinct human AAAP transporter family focussing upon SNAT5 (SLC38A5), a carrier with very different substrate specificity to PAT2.

### In SNAT5 (SLC38A5), A138 is a key molecular determinant of substrate specificity

The System N transporter (so-called, because it transports amino acids with nitrogen-containing side-chains) SNAT5, is a member of SLC38 and the AAAP (2.A.18) family (Figs. 1, 2, Table 1) [2, 52]. Although SLC38 and SLC36 are more closely related than to other mammalian SLC families (Figs. 1, 2), sequence identity is relatively low (e.g., 22%, PAT2 vs. SNAT5). SNAT5 functions differently from PAT2 being an electroneutral amino acid carrier involving Na^+^ cotransport and H^+^ efflux [41, 52]. SNAT5 prefers serine and amino acids with longer side-chains (e.g., asparagine and glutamine) but only interacts weakly with amino acids with shorter side-chains (e.g., alanine and glycine) [40–42, 52]. SNAT5 and PAT2 thus exhibit distinctive, almost opposing, substrate selectivity.

**Fig. 7:**
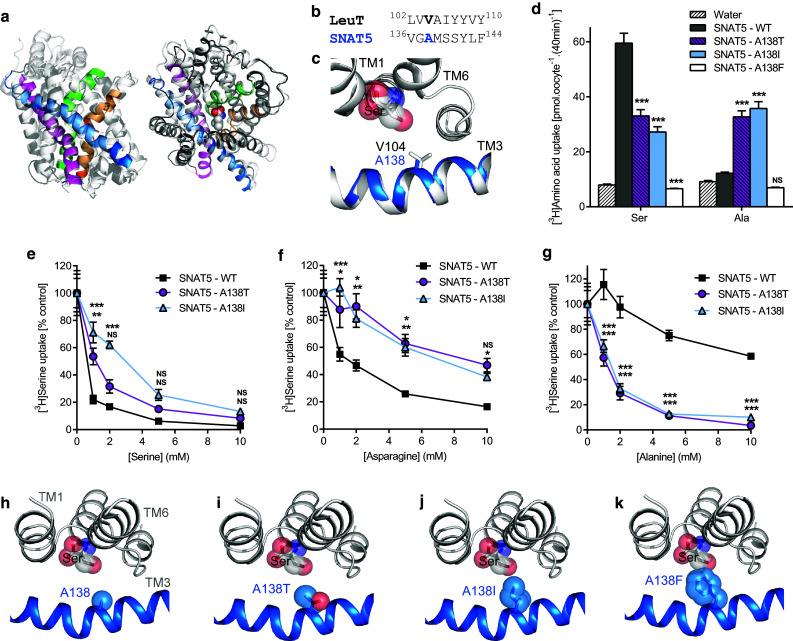
Mutation of the LeuT V104 equivalent residue (A138) in the System N transporter SNAT5 (SLC38A5) has a striking effect on substrate selectivity. **a** Human SNAT5 modelled against the crystal structure of LeuT (2A65). TM1-TM10 of LeuT are coloured light grey. The substrate-binding pocket of SNAT5 is predicted to be formed by TM1 (green), TM3 (blue), TM6 (orange), and TM8 (magenta). The remaining six SNAT5 TMs modelled are coloured dark grey (left-hand figure, within the plane of the membrane; right-hand figure, orientation above the plane of the membrane). For clarity, an extracellular loop (residues 227–247) in SNAT5 has been omitted due to a lack of predicted structural homology to LeuT. **b** Partial HHPred alignment of TM3 in LeuT and SNAT5. **c** Crystal structure of LeuT (light grey) with the predicted structure of SNAT5 TM3 superposed (blue). The side-chains of V104 (LeuT) and A138 (SNAT5) are shown as sticks. Serine is shown as a substrate (spheres) and was inserted into the binding pocket upon the leucine backbone using PyMOL. **d** Serine and alanine (both 50 μM) uptake (at pH 8.5, Na^+^-containing solution) were measured in wild-type SNAT5 (SNAT5-WT) and the SNAT5 mutants A138T, A138I, A138F. *n* = 20; NS, *P* > 0.05; ***, *P* < 0.001 vs. SNAT5-WT (ANOVA, Tukey’s multiple comparisons test). **e**–**g** Serine uptake (50 μM, pH 8.5, Na^+^) by SNAT5-WT, SNAT5-A138T, and SNAT5-A138I, measured in the presence of **e** unlabelled serine, **f** asparagine, or **g** alanine (all 0–10 mM). Data are expressed as % control (absence of competitor) after the subtraction of uptake into water-injected oocytes. *n* = 9–10; NS, *P* > 0.05; *, *P* < 0.05; **, *P* < 0.01; ***, *P* < 0.001 vs. SNAT5-WT (ANOVA, Tukey’s multiple comparisons test) whereby the upper symbols relate to SNAT5-A138I and the lower symbols to SNAT5-A138T. **h**–**k** Predicted substrate-binding pocket of SNAT5 modelled on LeuT with serine as a substrate. The side-chains of A138 (TM3), A138I and A138F are represented by light blue spheres (with the oxygen atom of threonine in red). Substitution of A138 with residues with sequentially larger side-chains [threonine, A138T (**i**); isoleucine, A138I (**j**); phenylalanine, A138F (**k**)] results in a concomitant decrease in the volume of the predicted substrate-binding pocket which reduces affinity for serine (**i**, **j**) (and asparagine) or abolishes transport (**k**)

Comparison of a human SNAT5 homology model with the outward-occluded substrate-bound crystal structure of LeuT (Fig. 7a) reveals that SNAT5 A138 occupies the equivalent position to LeuT V104 (and thus PAT2 F159) (Fig. 7b, c). Serine is transported well by SNAT5, whereas alanine is transported poorly (Fig. 7d) [41]. In a reversal of the protocol used with PAT2 (Fig. 5), SNAT5 residue 138 was mutated and systematically increased in size from alanine to phenylalanine (90.0–191.9 Å^3^) [50], producing A138T, A138I, and A138F. Reducing the SNAT5-binding pocket volume decreases serine transport in A138T and A138I and abolishes transport in A138F (Fig. 7d). The reduction in affinity for serine and asparagine (Fig. 7e, f) in A138T and A138I indicates that they do not fit as well within the smaller binding pocket. In contrast, the A138T and A138I mutants gain function and become excellent alanine transporters (Fig. 7d) with much improved affinity compared to SNAT5 (Fig. 7g). The affinities for serine and alanine change, such that SNAT5 favours serine, the smaller binding pocket of A138T appears to take both substrates with similar affinity, whereas the even smaller pocket of A138I prefers alanine (Fig. 7e–g). Predicted changes in the binding pocket are visualised in Fig. 7h–k.

## Discussion

In all forms of life, transmembrane transfer of nutrients, micronutrients, and excretory products is crucial to enable homeostasis, adaptation, and optimal cellular performance. Amino acid carriers are numerous in nature (see TCDB) [1], possess distinct functions, and exhibit dramatic differences in substrate specificity. Each cell type expresses a unique array of amino acid carriers. For example, the mammalian small intestinal epithelial cell expresses at least seven amino acid transport systems at the luminal surface, eight distinct mechanisms at the basolateral membrane, with many others being expressed in intracellular organelles. More than half of the 71 human LeuT-fold carriers demonstrate amino acid transport with that number likely to rise as orphan transporters are characterized functionally. Thus, the LeuT-fold is particularly efficient at transmembrane movement of amino acids. Each of the 37 human LeuT-fold amino acid carriers characterized thus far demonstrates a unique selectivity, varying greatly in substrate specificity and relative affinity. The heterogeneity in selectivity, and the currently limited understanding of its underlying molecular basis, was the motivation for this study. Rather than focussing simply on well-conserved residues, we thought it judicious to seek evidence for conservation of a discrete position within the LeuT-fold with the capacity to influence substrate recognition across the APC superfamily.

The properties (spatial, steric, chemical, and electrical) of the binding pocket of each transporter are determined by main-chain hydrogen bonding partners in the unwound regions of TM1 and TM6, along with the side-chains of various residues contained primarily within TM1, 3, 6, and 8 [18]. The principal means by which proteinogenic amino acid substrates are differentiated is by side-chain recognition. The side-chain of the V104 residue forms part of the hydrophobic pocket within the LeuT binding site and makes van der Waals’ contacts with the aliphatic substrate side-chain [3]. We hypothesised that the conserved function of LeuT V104, and of different amino acid residues occupying the equivalent site in other APC superfamily transporters (Table 1), is to shape the binding pocket and thus influence substrate selectivity.

To test this hypothesis, we studied exemplar mammalian carriers from the AAAP (2.A.18) family (within the APC superfamily) (Figs. 1, 2, Table 1) that are distinct in function, substrate specificity, and tissue expression [29, 40, 41, 52]. The SLC36 carrier, PAT2, is expressed at the human renal proximal tubular brush-border membrane where it reabsorbs glycine, alanine, and proline [44]. Defective PAT2 function contributes to iminoglycinuria (OMIM 242600) and hyperglycinuria (OMIM 138500) [44]. Other SLC36 carriers play roles in dietary amino acid uptake and in mTORC1 modulation [29, 51]. The SLC38 member SNAT5, which, in contrast, transports amino acids with larger side-chains (glutamine, asparagine, and serine), contributes to hepatic glutamine influx for protein synthesis, and glutamine efflux from astrocytes to support neurotransmitter recycling [52]. Other SLC38 carriers are important in amino acid transport in the brain, kidney, small intestine, placenta, and skeletal muscle [52]. In this investigation, sequential mutation of the equivalent residue to LeuT V104 in both PAT2 (F159) and SNAT5 (A138) allowed titration of substrate specificity based upon amino acid side-chain length (Figs. 3, 4, 5, 6, 7).

The equivalent residue to F159 is conserved within mammalian SLC36 transporters but varies in the arthropod expansion. The model arthropod carrier CG1139 (which is important in fly growth) [49] shares many functional characteristics with mammalian PATs but notably transports amino acids with larger side-chains, consistent with having an isoleucine rather than phenylalanine at position 149 [the LeuT V104-equivalent (Figs. 2 3, 4, 5)]. In PAT2-F159I, substrate selectivity broadened to become more CG1139-like with the key-determining factor being the space available within the hydrophobic-binding pocket (Figs. 4 and 6). Mutation to F159T and F159C decreased residue size, resulting in further changes in substrate selectivity, consistent with an increase in binding pocket volume enabling access and translocation of amino acids with longer side-chains (Fig. 5). Notably, a threonine is present at the equivalent position in the *Aedes aegypti* carrier AaePAT1 (Fig. 2, Table 1) which accepts amino acids with longer side-chains than alanine and glycine [53]. AaePAT1 is highly upregulated in the midgut following a blood meal and is responsible for amino acid uptake in the yellow-fever mosquito [53]. Similarly, a cysteine is found in the aphid *Acyrthosiphon pisum* transporter ApGLNT1 (Fig. 2, Table 1) (which sits at the bacteriocyte membrane at the symbiotic interface where it supplies glutamine [transported here by PAT2-F159C (Fig. 5)] to the proteobacterium *Buchnera aphidicola*) [54].

Thus, the homologous residue to LeuT V104 is a key determinant of substrate recognition in mammalian SLC36 (and SLC38) transporters and in more remotely related invertebrate carriers.

Furthermore, interpreting published data in the light of our work suggest that this site has a much broader and general significance for substrate selectivity across the APC transporter superfamily. Despite being only distantly related (Fig. 1), PAT2 (from the AAAP family) superposes on APC superfamily structures from the NSS, APC, and NCS1 families (Fig. 2). We find evidence for a role of this equivalent residue in substrate selectivity from functional and mutational studies of, mainly non-amino acid transporting, members of the SLC6, SLC7, and SLC12 families [22, 24, 55–62]. In SLC6 transporters from within the NSS (2.A.22) family (Fig. 1, Table 1), I172 in the serotonin transporter SERT, V152 in the dopamine transporter DAT (equivalent to V120 in the *Drosophila* DAT crystal structure, 4XP4), V148 in the noradrenaline transporter NET, and C144 in the creatine transporter CT1 are equivalent to LeuT V104 and are predicted to occupy sites close to the binding pockets. Even subtle mutations of these residues can modify substrate selectivity, affinity, and inhibitor (e.g., selective serotonin reuptake inhibitors) binding [22, 24, 55–59, 61]. These functional observations are confirmed in the crystal structures of human SERT and *Drosophila* DAT where the V104-equivalent residues define regions of the binding pockets associated with binding of substrates and antidepressants [12, 15, 45]. Similar observations are made in the APC (2.A.3) family which includes the structurally resolved AdiC and ApcT as well as the mammalian SLC7 transporters (Fig. 1, Table 1). Mutation of N133 in mouse LAT2 (slc7a8), by introduction of the LAT1 (SLC7A5)-equivalent residue, to produce N133S (corresponding to V104 in LeuT, N134 in human LAT2 and S144 in human LAT1), increases 3,3-diiodothyronine (T2) transport [62]. In the CCC (2.A.30) family, mutation of the V104 equivalent residue (A379) in the bumetanide-sensitive NKCC1 (SLC12A2) (Fig. 1, Table 1), a Na^+^/K^+^/2Cl^−^ cotransporter important in human fluid and electrolyte secretion and homeostasis, demonstrates that side-chain size was inversely related to ^86^Rb^+^ flux, and affinities for sodium and chloride were reduced compared to wild-type [60]. In the NCS1 (2.A.39) family (Fig. 1, Table 1), W117 in Mhp1 is conserved among all other members [5]. The indole ring of tryptophan forms a pi-stacking interaction with the hydantoin moiety of the Mhp1 substrate and presumably performs a similar function in other family members as all substrates contain ring structures [25]. Thus, the data reported here, supported by published data from a variety of eukaryote and prokaryote transporters, confirm that the residue occupying the equivalent position to LeuT V104 is important in determining substrate selectivity, in both amino acid transporters and other carriers, across the APC superfamily (Fig. 1, Table 1).

Amino acid transporters and other solute carriers (SLCs) are involved in many key physiological processes and, as such, are drug targets for treatment of numerous disease states [18, 20, 63]. In addition, SLCs are integral determinants of drug disposition as therapeutic agents can hijack transporters [64]. Thus, numerous prokaryotic and eukaryotic transporters are potential targets in overcoming disease-causing mutations, targeting disease-causing vectors, and improving drug delivery and agricultural yield. Realistically, we cannot determine the structure and function of all transporters throughout the kingdoms of life, particularly as the number of potential targets increases daily. Rather, accurate in silico modelling and predictive methods of function are something of a “holy grail”. Such methodologies are being used currently with some success in the identification of novel substrates for specific SLCs [65]. However, for rational approaches to novel drug design and treatment of disease states to be developed, and for such predictive modelling strategies to be successful, extensive knowledge and understanding of both structure and function of archetypal membrane transporters are required. Judging by the large number of distinct amino acid transporters in the APC superfamily (see TCDB) [1], the LeuT-fold appears particularly well adapted for the translocation of amino acids. Here, we show, through comprehensive functional studies, that a single divergent residue position is a principal molecular determinant of substrate specificity in LeuT-fold amino acid transporters. The V104-equivalent residue is an important piece of the puzzle which, along with future studies of both dynamic (functional) and static (structural) states, will enable construction of an accurate 3D functional map of the APC superfamily LeuT-fold.

### Electronic supplementary material

Below is the link to the electronic supplementary material.

Supplementary material 1 (XLSX 14 kb)
